# Cellular Factors Implicated in Filovirus Entry

**DOI:** 10.1155/2013/487585

**Published:** 2013-01-13

**Authors:** Suchita Bhattacharyya, Thomas J. Hope

**Affiliations:** ^1^University of Mumbai and Department of Atomic Energy-Centre for Excellence in Basic Sciences, Health Centre Building, Vidyanagari, Kalina, Santacruz East, Mumbai 400098, India; ^2^Department of Cell and Molecular Biology, Feinberg School of Medicine, Northwestern University, 303 East Superior Avenue, Chicago, IL 60611, USA

## Abstract

Although filoviral infections are still occurring in different parts of the world, there are no effective preventive or treatment strategies currently available against them. Not only do filoviruses cause a deadly infection, but they also have the potential of being used as biological weapons. This makes it imperative to comprehensively study these viruses in order to devise effective strategies to prevent the occurrence of these infections. Entry is the foremost step in the filoviral replication cycle and different studies have reported the involvement of a myriad of cellular factors including plasma membrane components, cytoskeletal proteins, endosomal components, and cytosolic factors in this process. Signaling molecules such as the TAM family of receptor tyrosine kinases comprising of Tyro3, Axl, and Mer have also been implicated as putative entry factors. Additionally, filoviruses are suggested to bind to a common receptor and recent studies have proposed T-cell immunoglobulin and mucin domain 1 (TIM-1) and Niemann-Pick C1 (NPC1) as potential receptor candidates. This paper summarizes the existing literature on filoviral entry with a special focus on cellular factors involved in this process and also highlights some fundamental questions. Future research aimed at answering these questions could be very useful in designing novel antiviral therapeutics.

## 1. Introduction

The *Filoviridae *family comprises of three genera:* Ebolavirus, Marburgvirus,* and *Cuevavirus* (tentative). These enveloped viruses are nonsegmented with negative-sense RNA and produce filamentous virions, which are pleomorphic in shape [1]. *Ebolavirus* has five known species: Zaire (EBOV), Sudan (SUDV), Reston (RESTV), Tai Forest (TAFV), and Bundibugyo (BDBV) while *Marburgvirus* has only one species: Marburg virus (MARV) [2–9]. EBOV and MARV and are known to be serologically, biochemically, and genetically distinct [10, 11]. 

The filoviral genome encodes seven structural proteins: envelope glycoprotein (GP), major matrix protein (VP40), nucleoprotein (NP), polymerase cofactor (VP35), replication/transcription protein (VP30), minor matrix protein (VP24), and RNA dependent DNA polymerase (L). In addition to this, EBOV also expresses a small, secreted, nonstructural glycoprotein (sGP) (see [12] for a comprehensive review).

Filoviruses are transmitted through contact with infected blood or body fluids [13] and can infect many cell types across different host species with lymphocytes being the notable exception [14, 15]. Although filoviruses are known to be pantropic, their preferred target cells include hepatocytes, dendritic cells, endothelial cells, macrophages, and monocytes (see [16] for a detailed review). Several species of fruit bats are suggested to act reservoirs for these viruses [17–21] and destroying these reservoirs could help to curtail the spread of these viruses. EBOV and MARV cause a fatal form of hemorrhagic fever [2, 6, 9, 12] and there are no vaccines or drugs currently available against them. Moreover, the US Centers for Disease Control and Prevention (CDC) has classified filoviruses as possible weapons for bioterrorism [22]. Therefore, these viruses need to be studied under Biosafety Level 4 conditions, which restricts the number of research laboratories that can work with these infectious viruses. 

Entry is the earliest step in the viral replication cycle and the filoviral entry process broadly involves the following steps: binding of the virus to its receptor(s)/attachment factors on the cell surface; uptake of the virus; intracellular trafficking of the virus in endosomes via clathrin, macropinocytic and/or caveolae-mediated endocytic pathways; viral fusion and release of the nucleocapsid into the cytoplasm. Earlier reports investigating various steps of the entry process have yielded conflicting results with various studies implicating or refuting the involvement of different cellular factors and endocytic pathways in this process. This paper summarizes the key findings underlying the various steps involved in filoviral entry with a special focus on the cellular factors implicated in this process and also discusses some unresolved issues in this field. 

## 2. The Filoviral GP Mediates Entry into Target Cells

The filoviral GP is the only protein present on the virus surface and facilitates receptor binding as well as fusion of the virus envelope with the host cell membrane [23]. GP is a type I transmembrane glycoprotein encoded by the fourth gene from the 3′ end of the genome [24], and is expressed as homotrimers, which form spikes on the surface of virus. Folding and assembly of EBOV GP trimers occurs independently of other viral proteins [25]. 

GP is expressed as a precursor protein that is post-translationally cleaved by a cellular proprotein convertase furin into GP1 (140kD) and GP2 (26kD) [26], which are linked by disulfide bonds. The GP1 subunit contains the receptor binding site and a heavily glycosylated mucin-like region (MLR), which facilitates viral attachment to target cells but is not required for viral entry *in vitro *[27]. The MLR also contains several epitopes, which are recognized by anti-GP antibodies to facilitate antibody-dependent enhancement of filoviral infection *in vitro* [28–30]. Furthermore, the crystal structure of EBOV GP demonstrates that the receptor binding site of GP1 is masked by a glycan cap and the MLR and therefore, removal of these regions could perhaps expose additional sites required for receptor/cofactor binding [31, 32]. The GP2 subunit contains two heptad repeat regions, which facilitate assembly of GP into trimers, a transmembrane anchor sequence, and the fusion loop [25, 33]. In MARV GP, the putative fusion domain is located 91 amino acids from the furin cleavage site [34]. The carboxy (C) terminus region of EBOV GP and MARV GP is very homologous and contains seven highly conserved cysteine residues, is high in proline content and has a short hydrophilic tail [24].

Despite the extensive homology at the C terminus, EBOV GP and MARV GP also exhibit several important distinctions. EBOV GP and MARV GP only share 31% identity in their amino acid sequence [35] and do not cross-react serologically [5]. Also, MARV GP is synthesized as a 170kD protein, which is encoded by a single open reading frame (ORF) [24, 36]; while EBOV GP is encoded in two ORFs and expression of the full-length GP occurs by transcriptional RNA editing [4]. 

## 3. Cellular Plasma Membrane Components Involved in Attachment and Uptake of Filoviruses 

Given the broad tissue tropism and host range of filoviruses, it was believed that the receptors of these viruses are ubiquitously expressed in most cells. Subsequently, beta 1 integrins [37] and several lectins such as DC-SIGN, DC-SIGNR, L-SIGN, and hMGL were shown to be involved in filovirus entry [38–42]. Matsuno and colleagues have demonstrated that the efficiency of C-type lectin mediated MARV entry differs between different strains [43] and that these lectins are not functional receptors for filoviral entry [44]. The role of another ubiquitous cellular factor folate receptor alpha in filoviral entry has been implicated and refuted by different groups [45, 46]. Two reports have suggested that the TAM family of receptor tyrosine kinases comprising of Tyro3, Axl and Mer are employed by filoviruses for entry [47, 48]. A more extensive analysis by Brindley and coworkers demonstrated that while Axl facilitated viral attachment and macropinocytic uptake of EBOV in several cell lines and primary cells, it did not bind to GP directly and hence is not a receptor for EBOV [49].

EBOV GP and MARV GP were initially suggested to bind to distinct cell surface residues for entry [14] and were also speculated to use different receptors for internalization into diverse cell types [50]. However, it is now known that these viruses bind to a common receptor [51–53]. 

Recently, T-cell immunoglobulin and mucin domain 1 (TIM-1) was reported to be a common receptor for EBOV and MARV [54]. TIM-1 is also known to bind to phosphatidyl serine, which is exposed on the surface of apoptotic cells and thereby facilitates phagocytosis of these cells [55]. Since viruses such as influenza are known to trigger expression of phosphatidyl serine on the surface of infected cells [56], it is possible that filoviruses also trigger expression of phosphatidyl serine on the surface of infected cells, which could then bind to TIM-1 and thereby facilitate viral uptake. Interestingly, TIM-1 is not expressed in macrophages and dendritic cells [57], which are the primary target cells of filoviral infection. Therefore, it is also possible that TIM-1 is merely one of many cellular factors that facilitate filoviral entry. The detailed mechanisms governing the interactions between filoviral GP and these cellular factors remain to be understood.

## 4. Cytoskeletal Components Involved in Filoviral Entry 

The involvement of cytoskeletal proteins in EBOV entry has been widely reported. Using pseudotyped virus, Yonezawa and coworkers showed that microtubules and microfilaments are required for EBOV entry [58]. Similarly, Ruthel and colleagues demonstrated that the EBOV matrix protein VP40 directly associates with microtubules [59]. Several studies have also demonstrated the involvement of actin and actin regulatory factors in EBOV entry [60–62]. Using fluorescently labeled EBOV, Saeed and coworkers showed that phosphoinositide-3 kinase (PI3K), Akt, and Rac1 are required for entry [63]. Using WT Zaire EBOV, Kolokoltsov and colleagues demonstrated a requirement of calcium/calmodulin kinase (CAMK2) in entry [64]. All these studies also support the role of macropinocytosis in EBOV entry.

## 5. Involvement of Clathrin, Macropinocytosis, and Caveolae Endocytic Pathways in Filoviral Entry

Using chemical inhibitors to block endocytosis, several groups have shown that filoviruses are endocytosed in a pH-dependent manner [14, 65–67]. Clathrin, macropinocytic, and caveolae-mediated endocytic pathways have all been implicated to be involved in filoviral entry. A few studies have also reported the concomitant use of multiple endocytic pathways in filoviral entry. However, the relative contribution of each of these endocytic pathways in filoviral entry into different cell types is still unclear. 

Using wild type as well as pseudotyped viruses, we and others have shown that filoviruses use clathrin-mediated endocytosis as an entry pathway [66–69]. We also performed a comprehensive analysis of the clathrin pathway using HIV pseudotyped with EBOV GP or MARV GP and found that filoviruses have a common requirement for several cellular factors of this pathway including clathrin heavy chain (CHC), phosphatidylinositol binding clathrin assembly protein (PICALM), epsin1, intersectin 1, dynamin 2, NUMB, low density lipoprotein receptor adaptor protein 1 (LDLRAP1), inositol polyphosphate phosphatase-like 1 (INPPL1), RALBP1-associated Eps domain containing 1 (REPS1), and RALBP1-associated Eps domain containing 2 (REPS2). Interestingly, while EBOV GP mediated entry was found to require Eps15, AP-2, and DAB2; MARV GP mediated entry was independent of these cellular factors and instead required Arrestin, beta 1 (ARRB1) [68]. This differential requirement for key components of the clathrin pathway in EBOV GP versus MARV GP mediated entry could perhaps be attributed to the differences in the composition of the GPs of these two viruses or the usage of additional cellular factors/coreceptors during entry.

Numerous groups have also described a role of macropinocytosis in EBOV entry [47, 60, 61, 69, 70]. Using biologically contained virions and virus-like particles (VLPs), Nanbo and colleagues showed that EBOV virions co-localize with sorting nexin (SNX) 5, which is a constituent of macropinosomes [70]. Hunt and colleagues demonstrated that Axl enhances macropinocytic uptake of EBOV [47]. Other cellular factors that were implicated in EBOV entry via macropinocytosis include p21-activated kinase (Pak1), ADP-ribosylation factor 6 (Arf6), C-terminal-binding protein 1 (CtBP1), Protein kinase C (PKC), and Phospholipase C (PLC) [47, 60, 61]. The role of dynamin 2 in macropinocytic entry of filoviruses was implicated and refuted by different groups [60, 61, 70]. Also, macropinocytic uptake of EBOV was shown to be independent of viral morphology [61]. However, the role of filoviral morphology in entry by clathrin and caveolae pathways has not yet been established.

A few reports have also demonstrated that filoviruses can simultaneously use multiple endocytic pathways for entry [47, 66, 69] and it was suggested that perhaps the virus preferentially chooses one pathway over another based on the type of target cells or receptors used [50]. 

 Although lipid rafts and membrane cholesterol were shown to be required for filoviral entry [58, 71], there are conflicting reports on the role of caveolae that are composed of membrane cholesterol, in filoviral entry with different studies implicating [47, 66, 72] or refuting [45] the involvement of caveolae in filoviral entry. 

Therefore, future studies examining the relative contribution and preference of each of these endocytic pathways in filoviral entry into target cells would prove insightful.

## 6. Endosomal Constituents Involved in Filoviral Entry

Studies examining the trafficking of filoviruses have revealed that after entry, the virus trafficks from early to late endosomes/lysosomes. Using GFP-labeled virions and VLPs, Saeed and colleagues have demonstrated that EBOV trafficks from EEA1 and Rab5-positive early endosomes to Rab7-positive late endosomes in HEK293T and Vero cells [60]. Similarly, Nanbo and coworkers have shown using biologically contained virions and VLPs that EBOV localizes to Rab7-positive late endosomes in Vero cells [70]. 

Several studies in Vero and Jurkat cell lines as well as mouse embryonic fibroblasts (MEFs) from cathepsins B and L deficient mice have demonstrated that proteolytic cleavage of EBOV GP by these lysosomal cysteine proteases removes the glycan cap and MLR of GP1 to produce a stable GP intermediate, which is necessary for infection [66, 73–75]. In contrast, Martinez and colleagues have reported that cathepsin L is not required for EBOV entry into human dendritic cells, which are one of the primary target cells of filoviral infection [76]. Moreover, a recent study by Misasi and coworkers showed using Vero and MEF cell lines that Zaire and Tai Forest species of EBOV require cathepsin B, while Sudan and Reston species as well as MARV do not [77]. Hence, the role of cathepsins B and L in filoviral entry into different cell types is not completely understood.

Recent reports have demonstrated that the endosomal membrane protein Niemann-Pick C1 (NPC1) can directly bind to EBOV GP and is an intracellular receptor for filoviruses [78–80]. These studies point towards the interesting possibility that cell surface receptors such as TIM-1 and endosomal receptors such as NPC1 perhaps act in concert with each other to facilitate filoviral entry. 

## 7. Cytosolic Cellular Factors Involved in Filoviral Entry

Several cytosolic factors were shown to be required for filoviral entry. Using EBOV GP pseudotyped virus, Yonezawa and colleagues have demonstrated that TNF-*α* enhances viral entry and fusion [58]. Similarly, using EBOV GP pseudotyped virus, Quinn and coworkers have showed that Rho B and C are required for EBOV entry [62]. Future studies investigating the involvement of additional cytosolic factors and the signaling pathways triggered by them to facilitate filoviral entry would be very useful.

## 8. Intracellular Factors Involved in Fusion and Release of Filoviral Nucleocapsid into the Cytoplasm

After GP1 is cleaved by the host cysteine cathepsins, the cleaved GP binds to NPC1 [78–80] and undergoes a series of conformational changes resulting in the refolding of GP2 into a six-helix bundle and insertion of its fusion loop into the host membrane. Viral membrane fusion results in the release of NP, VP35, VP30, L, and the RNA genome into the host cell cytoplasm. The cellular factors and molecular mechanisms governing the different steps of the fusion process are not clearly understood. 

## 9. Implications of Using a Common Cellular Receptor for Entry

Since filoviruses are suggested to bind to a common cellular receptor and yet can enter via multiple endocytic pathways, it is possible that these viruses require different coreceptor(s) and/or different processed or modified forms of the same primary receptor or coreceptor(s) for entry. Also, the involvement of cell surface receptors such as TIM-1 as well as endosomal receptors such as NPC1 in filoviral entry suggests that filoviruses utilize multiple receptors at various stages of the entry process. Future studies dissecting the interaction of filoviral GP with these receptors could be very insightful.

## 10. Therapeutic Implications 

 Small molecule inhibitors of NPC1 were shown to inhibit EBOV infection [78]. Hence, future research aimed at designing small molecule inhibitors of TIM-1 could be very useful for therapeutic purposes. Since TIM-1 can facilitate phagocytosis [55], specific inhibitors of phagocytosis can also be explored as potential therapeutic candidates. Additionally, several receptor tyrosine kinase inhibitors are already being used for treatment of numerous cancers [81–84] and therefore, specific inhibitors of TAM receptors could also be developed as anti-filoviral drug candidates. 

## 11. Future Directions

**Figure 1:**
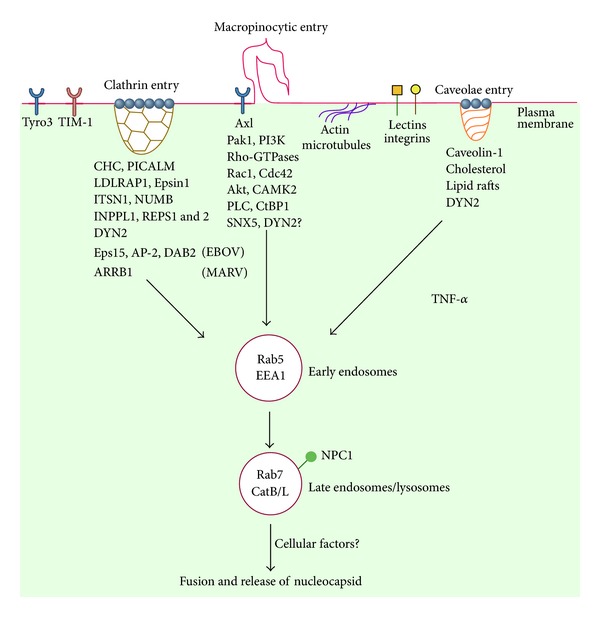
Schematic representation of cellular endocytic pathways and factors implicated in filovirus entry.

The mechanisms governing filoviral entry are not completely understood although recent studies have identified several cellular factors, which play critical roles in this process.  Figure 1 summarizes our existing knowledge on filoviral entry and the key cellular factors implicated in this process. However, there are several important pending questions the answers to which will greatly enhance our understanding of this field and also promote development of new avenues of therapy.

Understanding the detailed interactions of filoviruses with their cellular receptors/entry factors would be very useful in designing effective strategies to block these interactions. Since cellular factors are fixed targets, they are ideal candidates for development of effective broad-spectrum antiviral therapeutics. Therefore, it would be important to investigate the following broad issues.

- How do endosomal receptors such as NPC1 interact with cell surface receptors such as TIM-1 to facilitate viral entry? What are the molecular mechanisms governing the interactions of filoviral receptors with each other? Do the same residues of Filoviral GP bind to all the putative filoviral receptors? How do the filoviral receptors interact with key components of endocytic pathways to participate in filoviral entry?
- Do EBOV and MARV require any additional receptors and coreceptor(s) for entry? If so, are these receptors and coreceptor(s) conserved between the two viruses? Is the requirement for receptors and/or coreceptors cell type specific? Does differential expression of receptors/coreceptors on different cell types play a role in determining the preference for one endocytic pathway over the other? What are the molecular mechanisms governing the interactions of filoviral GP with its cellular receptors/coreceptors that enables filoviruses to exhibit broad tissue tropism and host range?
- What are the molecular mechanisms governing the induction of conformational changes in GP downstream of GP-NPC1 binding, to drive membrane fusion and release of the viral nucleocapsid into the cytoplasm? Which cellular factors play a role in this process?

Future research should be aimed at answering the above-mentioned issues, which could help to reveal as well as characterize the many intricacies involved in receptor binding, uptake, and entry of filovirus particles into target cells.
